# Pepper leaf disease recognition based on enhanced lightweight convolutional neural networks

**DOI:** 10.3389/fpls.2023.1230886

**Published:** 2023-08-09

**Authors:** Min Dai, Wenjing Sun, Lixing Wang, Md Mehedi Hassan Dorjoy, Shanwen Zhang, Hong Miao, Liangxiu Han, Xin Zhang, Mingyou Wang

**Affiliations:** ^1^ College of Mechanical Engineering, Yangzhou University, Yangzhou, China; ^2^ Nanjing Institute of Agricultural Mechanization, Ministry of Agriculture and Rural Affairs, Nanjing, China; ^3^ Faculty of Science and Engineering, Manchester Metropolitan University Manchester, Manchester, United Kingdom

**Keywords:** deep convolutional neural networks, crop disease recognition, GoogLeNet, real-time recognition, lightweight neural networks

## Abstract

Pepper leaf disease identification based on convolutional neural networks (CNNs) is one of the interesting research areas. However, most existing CNN-based pepper leaf disease detection models are suboptimal in terms of accuracy and computing performance. In particular, it is challenging to apply CNNs on embedded portable devices due to a large amount of computation and memory consumption for leaf disease recognition in large fields. Therefore, this paper introduces an enhanced lightweight model based on GoogLeNet architecture. The initial step involves compressing the Inception structure to reduce model parameters, leading to a remarkable enhancement in recognition speed. Furthermore, the network incorporates the spatial pyramid pooling structure to seamlessly integrate local and global features. Subsequently, the proposed improved model has been trained on the real dataset of 9183 images, containing 6 types of pepper diseases. The cross-validation results show that the model accuracy is 97.87%, which is 6% higher than that of GoogLeNet based on Inception-V1 and Inception-V3. The memory requirement of the model is only 10.3 MB, which is reduced by 52.31%-86.69%, comparing to GoogLeNet. We have also compared the model with the existing CNN-based models including AlexNet, ResNet-50 and MobileNet-V2. The result shows that the average inference time of the proposed model decreases by 61.49%, 41.78% and 23.81%, respectively. The results show that the proposed enhanced model can significantly improve performance in terms of accuracy and computing efficiency, which has potential to improve productivity in the pepper farming industry.

## 1 Introduction

Pepper is almost an indispensable crop in life and is closely related to human obesity rate cardiovascular disease (Spence, 2019). With the rapid growth of the global population, the demand for peppers has been growing. Diseases such as pepper powdery mildew, pepper anthracnose, and pepper white spot disease are the main factors affecting the yield and quality of pepper (Gu et al., 2021). Pepper leaf diseases are usually the most direct manifestation of early crop growth problems. Accurate and rapid recognition of pepper leaf diseases is essential for promptly identifying growth issues and enabling accurate prevention and control measures. The conventional approach based on visual inspection and human experience for recognizing pepper leaves is subjective and time-consuming and costly. Therefore, there is a pressing need to develop a precise, fast, and convenient approach for detecting pepper leaf diseases.

In order to solve the problem mentioned above, classical machine learning methods like K nearest neighbor (KNN) (Xie et al., 2017), support vector machine (SVM) (D.Pujari et al., 2016), random forest (RF) (Gold et al., 2020), Naive Bayes (NB) (Mondal et al., 2017), artificial neural networks (ANN) (Wang et al., 2018) have been widely used in the field of crop leaf disease recognition. Despite existing works are encouraging, there are still limitations such as suboptimal performance (inaccuracy, computing efficiency) and lack of generalization capability. Moreover, the process becomes more complicated when dealing with huge leaf diseases dataset and many type of diseases, making the formal deployment and application of the models more difficult.

With the development of artificial intelligence theory, deep learning has been proposed to solve complex vision tasks. In the field of agriculture, different deep learning algorithms like Convolutional Neural Networks (CNNs), Gated Recurrent Units (GRU), and Long Short-Term Memory (LSTM) have been investigated for the recognition of the symptoms of major diseases that affect crops. In particular CNN, as one of the most promising techniques, has been successfully used in crop leaf disease recognition (Saleem et al., 2019). A variety of convolutional neural network models are closely integrated with recognizing crop leaf diseases (Ak et al., 2019; Klompenburg et al., 2020; Abade et al., 2021). After the deep learning model is built, it often needs to be transplanted into an external system. Therefore, while considering the recognition accuracy, the complexity of training should be as much as possible to make the model more lightweight (Mccool et al., 2017). However, achieving a high recognition rate often requires increasing the depth of the network, leading to the challenge of training complexity. Additionally, various activation functions, optimizers, and regularization methods have diverse effects on model training. Therefore, how to balance the network depth and training complexity and how to choose the appropriate activation function and optimizer for the network model has always been a difficult problem. It is worth noting that GoogLeNet has been widely used in different domains including crop diseases (Li et al., 2020; Yang et al., 2023). Unfortunately, it has some disadvanatges, for instance, GoogLeNet has complex network architecture and large model size. Therefore, to address these challenges, built on GoogLeNet architecture, this work has proposed a new deep convolution neural network model, i.e., an enhanced lightweight GoogLeNet (GoogLeNet-EL) model for accurate and efficient detection of multiple pepper diseases. The main contributions of this work include the following aspects:

(1) From the model perspective, an enhanced lightweight CNN model is proposed to accurately recognize pepper leaf diseases. Different from the existing GoogLeNet, we have introduced a spatial pyramid pooling (SPP) structure to enhance the model's learning ability of image features at different scales. In addition, we have also introduced a compressing method to optimize the network depth and width of the Inception module, leading to increased computing efficiency. The integration of compression and SPP greatly improves the model performance in terms of both accuracy and computing.
(2) From the data perspective, in order to meet the training data requirements for deep learning, we have collected a total of 9183 images to evaluate the performance of the proposed model, containing 6 types of pepper leaf diseases including pepper scab, pepper powdery mildew, pepper anthracnose, pepper white spot disease, pepper blight, and pepper botrytis cinerea.
(3) From the practical application of the model perspective, the proposed model has significant advantages in recognition accuracy and computing performance of pepper leaf diseases on a limited computing platform, which is beneficial to the further deployment in pepper plant in large fields. The improved performance can improve efficiency and reduce cost input.

The remainder of the paper is organized as follows. Section 2 introduces the existing related work. Section 3 describes the dataset acquisition, preprocessing, and collation regarding pepper leaf diseases, and then introduces the methodology required to accomplish this task of pepper leaf disease recognition along with related concepts and the proposed approach. Section 4 conducts the experimentations designed to investigate the factors that affect the performance of the proposed approach and the comparison test with other methods. Finally, the conclusion is presented in Section 5.

## 2 Related work

### 2.1 CNNs on crop diseases detection

As for the recognition of pepper leaf diseases, accuracy is the central performance of the convolution neural network model. Many previous works on disease recognition of other crops have proven that these CNN-based methods could achieve high recognition precision (Abade et al., 2021). **Table 1** displays crop diseases recognition based on CNNs by some recent literature since 2018, and the crucial fields such as the CNN model used, the best accuracy, and the study object are included it.

**Table 1:** Crop diseases recognition based on CNNs.

Literature	CNN models	Best accuracy	Study object
Zhang et al. (2018)	AlexNet - GoogLeNet model	97.28%	tomato leaf disease
Hu et al. (2019)	CNN model	92.5%	tea leaf diseases
Li et al. (2019)	VGG model	98.33%	crop diseases
Singh et al. (2019)	multi-layer CNN model	98%	mango leaf diseases
Chen et al. (2020)	VGGNet model	91.83%	rice disease
Ji et al. (2020)	BR-CNNs model	85.28%	crop leaf diseases
Waheed et al. (2020)	DenseNet model	98.06%	corn leaf disease
Chen et al. (2021)	MobileNet-V2 model	99.13%	crop diseases
Gao et al. (2021)	ResNet model	98.54%	cucumber disease
Li et al. (2022)	CNN model	94.22%	apple disease
Liu et al. (2022)	CNN model	97.54%	buckwheat diseases
Wang (2022)	AlexNet model	96.26%	fragrant pear diseases
Yang et al. (2023)	GoogLeNet model	99.58%	rice leaf diseases
Yang and Liu (2023)	CNN model	96.24%	rice disease
Yu et al. (2023)	RNN model	99.54%	soybean leaf diseases

From **Table 1**, it can be easily observed that that these CNN-based methods have shown favorable recognition results in the field of different crops diseases detection. Thus, it is significant to investigate pepper leaf disease recognition based on their feature extraction method by training all kinds of convolution kernels.

In recent years, some researchers have focused on pepper leaf disease recognition by employing deep learning algorithms. Wu et al. (2020) investigated an integrated neural network based on CNN for automatic detection and severity assessment of pepper bacterial spot disease, yielding the best overall accuracy of 95.34%. Then, some deep learning models were presented to diagnose image-based hot pepper disease and pests using deep features based on transfer learning (Yin et al., 2020; Gu et al., 2021). Mathew and Mahesh (2022) used YOLOv5 to detect the bacterial spot disease in bell pepper plant from the symptoms which can be seen on the leaves taken from the farm. In addition, Mahesh and Mathew (2023) focused on the bacterial spot disease detected on the image of the bell pepper plant by using YOLOv3 and showed a mean average precision of 90%. Mustafa et al. (2023) proposed a five-layered CNN model for automatic detection of pepper bell plant disease utilizing leaf images, predicting the plant leaf as healthy or bacterial with 99.99% accuracy. Although the promising progress has been achieved, it is still challenging in large pepper planting fields. On the one hand, much research has been carried out in the lab and the dataset has been collected from benchmark dataset. There is only limited data set of pepper leaf diseases for real-time detection. On the other hand, compared with different crop diseases, pepper diseases recognition is much more difficult due to large intra-class similarity and small inter-class variance in pathological symptoms (Wu et al., 2020).

### 2.2 Model lightweight

In addition, CNN-based models tend to require relatively high computational resources. This poses a challenge for their practical implementation in agricultural production settings with limited resources. Therefore, it becomes essential to consider other performance indicators, such as memory requirements, training time, and recognition time, to ensure feasibility and efficiency in such contexts. There have been growing concerns about lightweight CNN models for crop diseases detection. Thakur et al. (2023) proposed a VGG-ICNN model with seven convolution layers by reducing the CNN model size to identify crop disease and it outperformed most of CNN models. Haque et al. (2022) presented a lightweight CNN architecture with two modified Inception modules to identify the severity stages of maydis leaf blight disease of maize. To reduce the parameters and computations of the existing pest detection methods, Cheng et al. (2022) addressed a lightweight crop pest detection method by simplifying YOLOv3. Bhujel et al. (2022) presented a lightweight attention-based CNN model to detect tomato leaf disease and it reduced network parameters and complexity compared to the standard ResNet50 model. By compressing the AlexNet structure, Xie et al. (2021) proposed the CarrotNet to reduce the model training time to half of the original, which can meet the defect identification of crops. Kamal et al. (2019) introduced the separable convolution to the model, which made the training parameters of the model 29 times less than that of VGG and 6 times less than that of MobileNet. Aiming at the problem of too many AlexNet parameters, Zhang et al. (2019) combined the dilated convolution and global pooling to identify 6 common cucumber diseases and achieved good results. In summary, the aforementioned lightweight models have shown promising performance. As the complexity of network structures has hindered their practical implementation in agricultural production, such as pepper planting in large fields, there is a pressing need to explore suitable lightweight structures based on CNN models.

### 2.3 Crop disease dataset

At present, the identification dataset of crop diseases mainly depends on public datasets such as Plant Village dataset, Plant disease recognition dataset, New Plant Diseases Dataset, and CVPR 2020-FGVG7. There is only limited pepper leaf disease data in these datasets. To better and more effectively identify pepper leaf disease, different classes of diseases should be added to public dataset. In addition, it is different from the pepper leaf disease detection dataset in real-time. The image information of pepper leaf for actual scenes is often complex and redundant, which could result in the problem that CNN deployed under general calculated performance is used for the low accuracy and slow recognition speed of pepper leaf diseases. The principal means is to improve the existing network structure. Although the accuracy has reached a reasonable level, few studies can weigh the accuracy of model recognition and model parameters (Kim et al., 2016). Therefore, to identify pepper leaf diseases, using pepper leaf disease dataset from real-world scenes and develop a network model structure with high recognition accuracy that facilitates easy model training and deployment is important, which can serve as a valuable reference for pepper production and planting enterprises and contributes to the sustainable development of the agricultural industry.

## 3 Materials and methods

### 3.1 The overview of the proposed method

**Figure 1:**
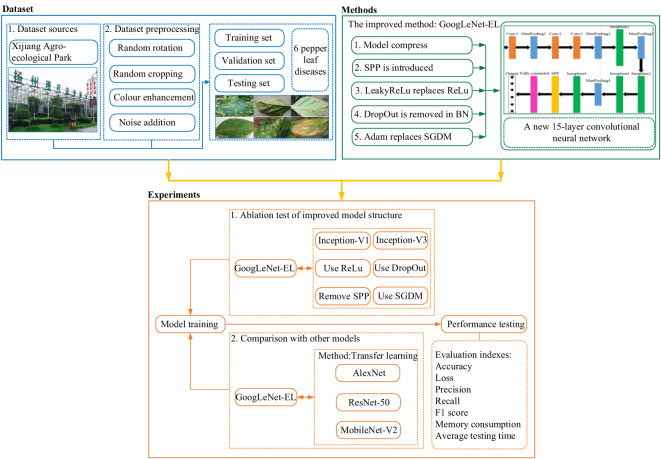
The overview process of the improved model.

As shown in **Figure 1**, a general overview of the improved CNN model based on GoogLeNet for pepper leaf disease recognition is described as follows. The first step is to use the current situation of pepper planting on the farm to build a dataset on six common diseases of pepper leaves. The next step is the introduction of methods to improve the model by investigating the GoogLeNet model, activation function, batch normalization layer, spatial pyramid pooling layer, and optimizer. The content is explained in detail in this section. Finally, our dataset is used for the improved model for training and experiment. The experiment is divided into two parts. The first test is a necessity test, which mainly examines the necessity of our proposed improvement aspects. The second test is the comparison with other models. The experimental test and result analysis are introduced in subsequent sections.

### 3.2 Dataset description

The dataset has an important influence on the accuracy of the approaches that implement the recognition of pepper leaf diseases. In this section, the investigated dataset was composed of RGB images. It was employed to train and test the models studied from two aspects: dataset source and dataset preprocessing. In the experiments, we used a total of 9183 images which contained 5669 original pepper leaf disease images and 3514 preprocessed pepper leaf disease images with various image augmentation.

#### 3.2.1 Dataset source

**Figure 2:**
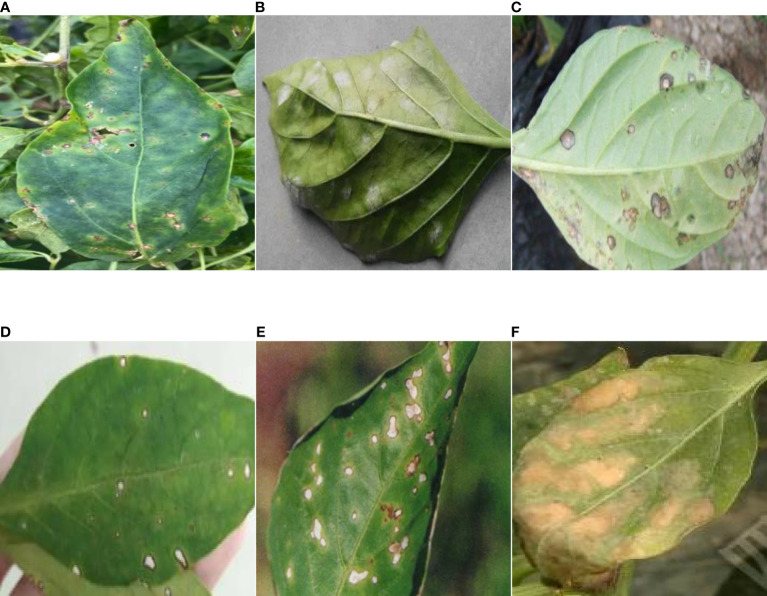
Related images of pepper leaf disease. **(A)** Pepper scab, **(B)** Pepper powdery mildew, **(C)** Pepper anthracnose, **(D)** Pepper white spot disease, **(E)** Pepper blight, **(F)** Pepper botrytis cinerea.

The data on pepper leaf diseases used in this paper is collected from Xijiang Agro-ecological Park, Yangzhou City, Jiangsu Province. Since the diseases are affected by seasonality, our dataset collection focuses on four different quarters in 2020, using a handheld camera to examine the diseased pepper leaves. We captured 5669 original pepper leaf disease images with annotation from agronomists. There are 6 classes of diseases, including pepper scab, pepper powdery mildew, pepper anthracnose, pepper white spot disease, pepper blight, and pepper botrytis cinerea. Some example images of pepper leaf diseases are shown in **Figure 2**.

#### 3.2.2 Dataset preprocessing

**Figure 3:**
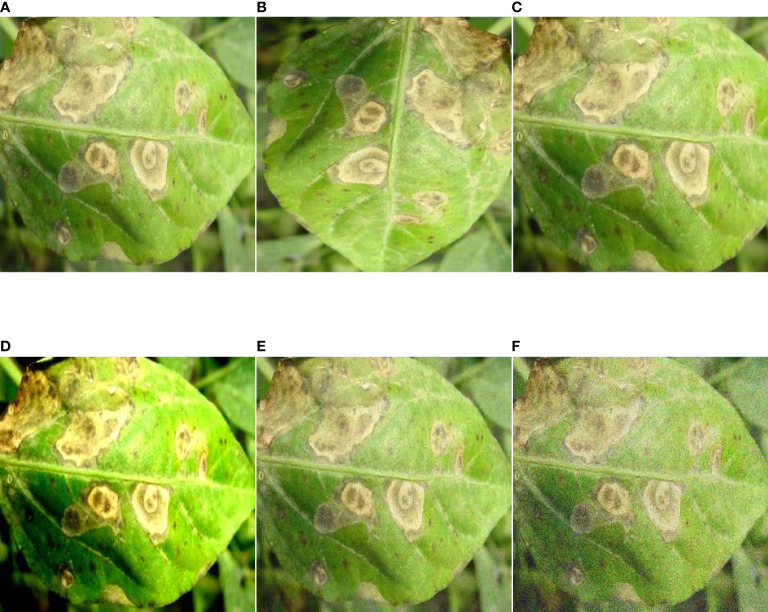
Image enhancement. **(A)** Original image, **(B)** 90-degree rotation, **(C)** Random crop, **(D)** Color enhancement, **(E)** Salt and pepper noise, **(F)** Gaussian noise.

Data augmentation is an essential means of balancing the number of samples and expanding the amount of data in deep learning technology. Overfitting can be effectively avoided through data augmentation, and the model's generalization ability can be improved. Common data enhancement methods include random rotation, random cropping, color enhancement, and noise addition. Through the above data enhancement operation, 3514 augmentation images of pepper leaf disease are obtained. The detailed list of the dataset is shown in **Table 2**. An example of pepper image enhancement can be seen in **Figure 3**.

**Table 2:** The detailed list of the dataset.

No.	Category	Training set and validation set (piece)	
		Original images (5669 images)	Preprocessed images (3514 images)
1	Pepper scab	1023	1546
2	Pepper powdery mildew	898	1537
3	Pepper anthracnose	733	1539
4	Pepper white spot disease	992	1540
5	pepper blight	1021	1488
6	Pepper botrytis cinerea	1002	1533

### 3.3 The proposed GoogLeNet-EL model for recognition of pepper diseases

#### 3.3.1 GoogLeNet

**Figure 4:**
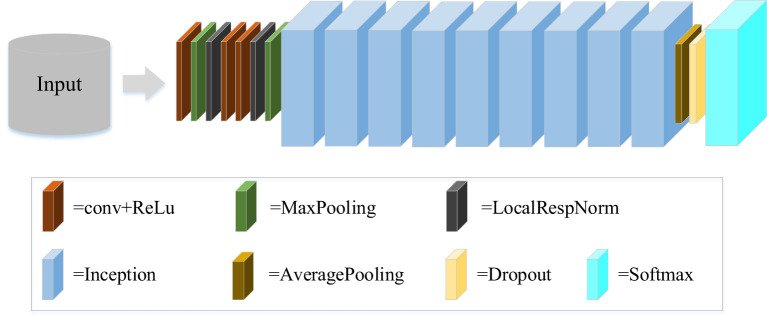
GoogLeNet structure.

GoogLeNet is a new structure of deep learning proposed by Christian Szegedy in 2014 (Szegedy et al., 2015), which combines the multi-scale idea and dimension reduction layers. GoogLeNet is a classical convolutional neural network, as shown in **Figure 4**. The original network of GoogLeNet consists mainly of three convolutional layers, nine Inception modules, and other components. The Inception module is the core component of GoogLeNet, including Inception-V1, Inception-V2, Inception-V3, and Inception-V4. Where, Inception-V1 is the basic module that other versions of the module use to improve network performance. Inception-V2 introduces Batch Normalization (BN) algorithm to reduce internal covariate shift. Compared with Inception-V2, Inception-V3 mainly introduces the idea of factorization in addition to BN algorithm. It can greatly reduce the amount of computation and further increase the depth of the network. When the network is deeper and wider, Inception-V4 is proposed to make it have a more unified inception structure. In recent years, GoogLeNet has been proven to perform well in many fields like scene recognition, medicine, and human pose estimation. In this study, from the perspective of basic model architecture construction and rapid feature recognition, we selected Inception-V1 and Inception-V3 used in this study.

Inception-V1 is a 22-layer network model, which is used to control arithmetic power under the existing datasets. The original structure of Inception-V1 includes a 1×1 convolution, a 3×3 convolution, a 5×5 convolution, and a 3×3 max pooling. Convolutions stacked in various ways increase the depth and width of the network. It extracts image features from multiple scales. However, the number of parameters is still large. To solve the problem of a large number of calculations, the complete structure of Inception-V1 is presented by adding three 1×1 convolutions. The structure can effectively reduce the number of feature channels and calculations. Thus, in our experiments, it is considered as the base model architecture and modified to generate a new network.

Inception-V3 is created by further optimizing the accuracy and calculation of Inception-V1. In terms of computational power, since the computational cost of a 5×5 convolution is 2.78 times of a 3×3 convolution, two 3×3 convolutions kernels are used instead of a 5×5 convolutions kernel. On this basis, the convolution kernel of n×n is decomposed into two convolution kernels, 1×*n* and *n*×1. When equivalent results are obtained, the calculation speed is greatly improved. The depth and width of the network are also increased, which is conducive to improving accuracy. Therefore, the idea regarding the factorization into small convolutions of Inception-V3 is used for the proposed deep convolutional neural network.

#### 3.3.2 Proposed GoogLeNet-EL

**Figure 5:**
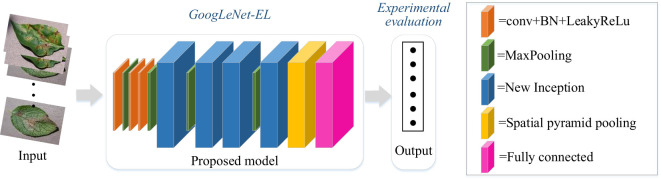
GoogLeNet-EL structure.

As mentioned earlier, GoogLeNet has shown competitive performance in dealing with image recognition and classification problems. Specifically, the use of the Inception module in GoogLeNet can reduce the number of parameters. Due to the large volume of the model and the great computational complexity, a valid lightweight network architecture is necessary. In the section, building on and extending on GoogLeNet with Inception-V3 as main neural unit, we have proposed the enhanced lightweight GoogLeNet-EL model. A compressing method is proposed to optimize the network depth and width of the Inception module, leading to increased computing efficiency. In addition, we have introduced a spatial pyramid pooling (SPP) to efficiently extract features at varies scales in the pooling layer. Furthermore, we have also optimized other components of GoogLeNet structure. Hence, an improved GoogLeNet structure diagram is proposed in **Figure 5** and the specific parameters are shown in **Table 3**. We compressed its model structure and only retained 4 Inception modules.

**Table 3:** Parameters of the improved GoogLeNet-EL model.

Layer name (Convolutional kernel size, number of convolutional kernels/step size)	Output size
Input layer	224×224×3
Convolutional layer(7×7, 64/2)	112×112×64
MaxPooling(3×3, /2)	56×56×64
Convolutional layer(1×1, 64/1)	56×56×64
Convolutional layer(3×3, 192/1)	56×56×192
MaxPooling(3×3, /2)	28×28×192
Inception1	28×28×480
MaxPooling(3×3, /2)	14×14×480
Inception2	14×14×512
Inception3	14×14×832
MaxPooling(3×3, /2)	7×7×832
Inception4	7×7×1024
SPP layer	7×7×1024
Fully connected layer	1×1×1024
Output layer	6

##### 3.3.2.1 Improved Inception module

**Figure 6:**
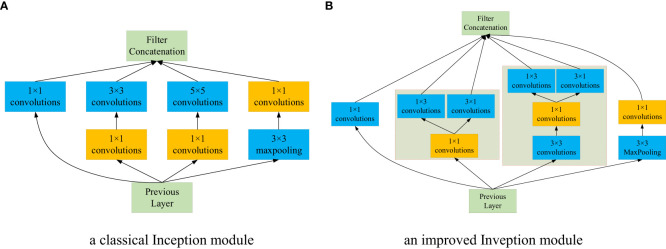
The structures of the Inception module and optimized Inception module. **(A)** a classical Inception module. **(B)** an improved Inveption module.

As shown in **Figure 6A**, a classical Inception module, i.e., Inception V1 was employed as the base module, which is comprised of 4 parallel layers. It can effectively reduce the number of feature channels and calculations. Despite competitive advantages, Inception V1 can be optimized to reduce the number of parameters. Based on the idea of factorization of Inception V3 (Szegedy et al., 2016), the convolution kernel of 5×5 is decomposed into two 3×3 convolution kernels. In addition, depth-wise separable convolution proposed by Chen et al. (2022) can reduce the computational complexity and compress the model size. It decomposes a depth-wise convolution and a 1×1 point-wise convolution, where the former can perform the filtering operation for each channel of the input feature map, and the later can carry out the combining operation. Owning to the lightweight advantage of depth-wise separable convolution, it can reduce the model size and improve the calculation speed without affecting the efficiency of feature extraction. Therefore, motivated by the above promising performance, we improved the architecture of the base module without a significant increase in parallel layers. In the second layer of Inception-V1, the 3×3 convolution was decomposed into two convolution kernels, 1×3 and 3×1; In the third layer of Inception-V1, the 5×5 convolution was replaced with two 3×3 convolution kernels, then one 3×3 convolution along with the original 1×1 convolution were substituted with 3×3 depth-wise and 1×1 point-wise convolutions, respectively; at last, the other 3×3 convolution is transformed with 1×3 and 3×1 convolutions. An improved Inception module is created in terms of Inception-V1, as shown in **Figure 6B**.

##### 3.3.2.2 The spatial pyramid pooling layer

**Figure 7:**
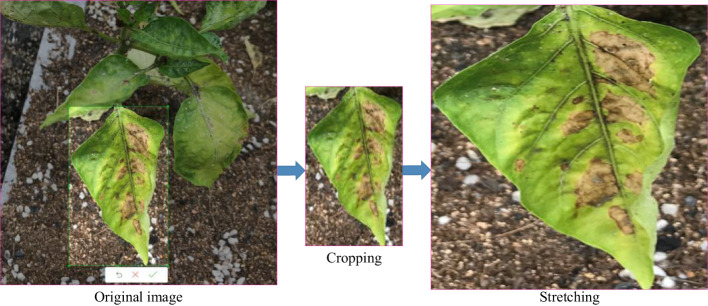
Cropping of pepper leaf disease image.

The input of CNN is fixed, but due to its nature, shooting angle, and other reasons, the size of our target image does not match the size of the fixed input. Taking the Pepper botrytis cinerea image (see **Figure 7**) as an example, the size of the target image we cut out is smaller than the actual size required by the network. The common method to deal with the problem is to crop and stretch the original image. However, the operation method could lead to the imbalance of the original scale of the target image size, resulting in image distortion. It can affect the accuracy of network training. Fortunately, spatial pyramid pooling (SPP) can effectively solve the problem (He et al., 2015).

**Figure 8:**
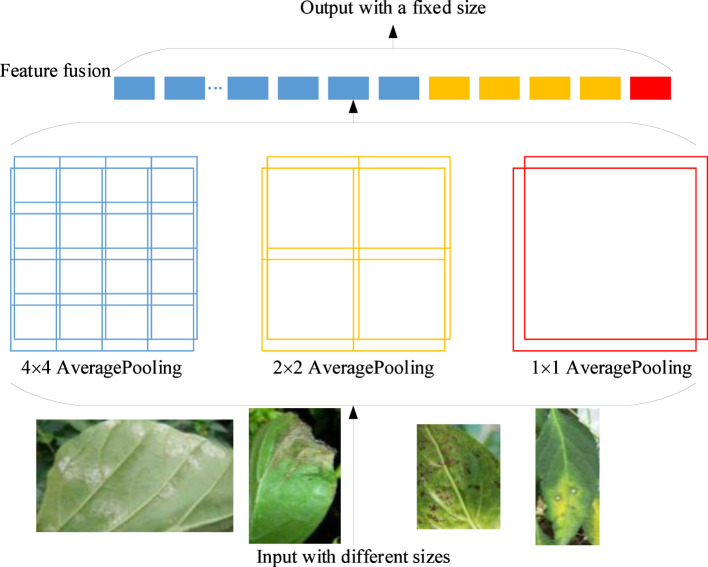
The network structure with SPP.

In order to better extract the feature of pepper leaf diseases, the SPP layer is added after the Inception4 module in our proposed model. There is no need to normalize the input of the GoogLeNet-EL model, saving much work. In this way, the original image features can be better preserved. The structure of SPP is shown in **Figure 8**. After Inception4, the average pooling of 4×4, 2×2, and 1×1 is used to divide the feature set into 16, 4, and 1 blocks. Finally, a 21-dimensional feature vector with a fixed size is obtained by fusing with features. Specifically, a feature multi-scale fusion technology is employed to effectively fuse features from different levels of the network. According to the network structure of GoogLeNet with Inception-V3, it has the ability to extract lower-level features (like edges and colors) and higher-level features (like deformity and necrosis). The feature information is first performed in terms of convolution operations with kernels of different on the input layer, which generates feature maps of different scales. Then, by using up-sampling and down-sampling operations, the scale of the feature maps is adjusted to be consistent. The final feature map can be obtained by effectively fusing these features and it has the richer and more comprehensive pepper leaf disease feature information. Therefore, any input can be converted into a feature vector with a fixed size, removing the limitation of input size for images of different scales.

##### 3.3.2.3 Other components optimizing the network structure of the proposed model

(1) Activation function selection. The non-linear output of the activation function enables CNN to distinguish the types of features effectively. ReLu, as a representative of the activation function, is widely used in convolutional neural networks due to its simple and efficient processing method. Its mathematical expression is as follows


(1) ReLu=max(x,0)

It is noted that ReLu itself is flawed, as shown in **Figure 8**. Although the processing mode that the output is set to zero whether the input variable *x<* 0 improves the calculation speed to a certain extent, it could lead to the failure to learn from some network features. When the backpropagation is carried out, the weights and bias function may not be updated, thus affecting the recognition accuracy of the model. In addition, the output of the ReLu is negative. It means that the saw-tooth problem caused by parameters and new directions occurs, which affects the training process.

**Figure 9:**
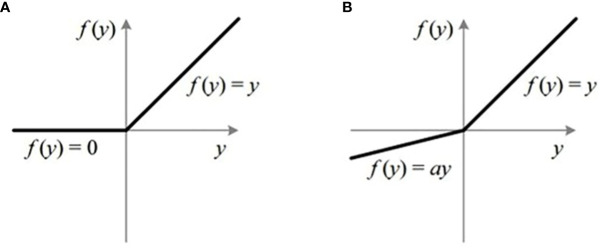
Activation function. **(A)** ReLu **(B)** LeakyRelu.


In order to deal with the above problem, we introduce one of its variants, LeakyReLu (see **Figure 9**). Different from ReLu, a small negative gradient is employed in LeakyReLu. In this way, LeakyReLu solves the problem of gradient disappearance and keeps learning network features. Moreover, the small negative gradient gives the activation function the option of negative numbers. This degree of differentiation is very important for the training of the model. Some studies show that LeakyReLu could replace ReLu as an activation function, and its performance is better than ReLu (Hichri et al., 2019; Wei et al., 2019). Therefore, we take LeakyReLu as the activation function of the network, and its mathematical expression is as follows

(2) LeakyReLu=max(x,∂x)

(2) DropOut and Batch Normalization. DropOut can solve the overfitting problem of GoogLeNet. It works by randomly discarding neurons with a certain probability. The probability value is usually set to 0.4 or 0.5, which is usually added to the last layers of the network. In forward propagation, every neuron disappears with a certain probability, which avoids the over-fitting of the model to a certain extent, thus improving the training speed. However, this method of discarding neurons with the standard probability is inconsistent with the response mechanism of the neural network, which has a negative impact on model training.

Batch normalization (BN) can accelerate deep network training by reducing internal covariate shift (Ioffe and Szegedy, 2015). When the data based on BN is used for the in-depth calculation in the network, the data distribution of each input is very stable, thus accelerating the convergence speed of the model. Hence the BN method is adopted in the improved model, which is introduced between each convolution and the activation function. In addition, since the mean and variance of each batch output are different, it brings random noise to the convolutional neural network, which has a regularization effect to a certain extent. BN has better performance than DropOut, which can be discarded (Garbin et al., 2020).

(3) Optimizer selection. The optimizer in CNN is mainly used to calculate the gradient in each round of training. It updates the parameters to minimize the loss. Therefore, it is essential to choose the right optimizer for the GoogLeNet-EL model. Commonly used optimizing approaches can be classified into three types in the optimizer: gradient descent algorithm, momentum algorithm, and adaptive learning rate optimization algorithm. In particular, stochastic gradient descent with momentum (SGDM) and Adam are two popular algorithms.

On the one hand, SGDM tends to generate better results in terms of fine-tuning parameters. However, the convergence speed is time-consuming when the gradient of SGDM is flat. Usually, SGDM can optimize the model to the extreme before the model goes live or the results are released. On the other hand, Adam has advantages for sparse data, and the convergence speed is fast. Adam combines the advantages of the momentum algorithm and the gradient L2 norm-based algorithm. It makes adaptive adjustments from the two angles of gradient mean and gradient square, such that the update of the gradient is not affected by gradient changes. In the experimental process of the model design, Adam can be used for rapid experimental optimization to verify the effect of the new model quickly. Many works using Adam as the optimizer of CNN have been proven to have good results (Bera and Shrivastava, 2020; Chang et al., 2020). Therefore, to improve the training speed of the GoogLeNet-EL, we choose Adam as the optimizer.

## 4 Experimental evaluation

### 4.1 Experimental platform and related hyperparameter settings

The experiments were carried out on a Windows desktop with a memory of 16GB, Intel(R) Core (TM) i7-9750H CPU @GHz, and NVIDIA GeForce GTX 1650. To avoid the impact of other parameters on the model performance test, we first trained GoogLeNet-EL at learning rates of 0.01, 0.001, and 0.0001. After 100 epochs, the final accuracies are 63.68%, 97.87%, and 94.72%, respectively. Thus, the learning rate of 0.001 is used uniformly in the follow-up test. Other related hyperparameters are set as follows: after 100 iterations, the accuracy and loss of model training are compared, and BatchSize is set to 220. The regularization coefficient *L*_2_ is set to 0.0001. Every 5 iterations are validated on the validation set, and a single GPU is used for training.

### 4.2 Performance metrics

The confusion matrix is used to evaluate the recognition performance of the model. The evaluation indexes include accuracy, recall, and F1 score. To further test the transplantation ability of the model in the real scene, average testing time is introduced to investigate the portability and applicability of different models. The relevant expressions are as follows


(3) $$Precision=\frac{TP}{TP+FP}$$

(4) $$Recall=\frac{TP}{TP+FN}$$

(5) $$F1=2\times \frac{Precision\times Recall}{Precision+Recall}$$

(6) $$Average\;testing\;time=\frac{\text{Testing time for all samples on the testing set}}{\text{The total number of samples on the testing set}}\times 100\%$$

Where *TP* is the number of positive samples predicted as positive samples, *FP* is the number of negative samples predicted as positive samples, and *FN* is the number of positive samples predicted as negative samples.

### 4.3 Experimental goals

To evaluate the performance of the proposed model, we have conducted a series of experiments as follows:

Experiment 1: ablation study on Inception module. We carried out ablation experiments on different inception modules. Performance tests of models with Inception-V1, Inception-V3, and our improved Inception were compared, including the accuracy, loss, training time, and memory requirements.

Experiment 2: ablation study on different components optimizing network structure of the proposed model. We focused on how different components optimize network structure of the proposed GoogLeNet-EL model. Based on the structure of the proposed GoogLeNet-EL, we have performed separately ablation studies on spatial pyramid pooling, activation function selection, DropOut, batch normalization, and optimizer selection to validate the effectiveness of our improved model. Here, besides the improved Inception module, the GoogLeNet-EL structure is mainly composed of LeakyReLu function, batch normalization, spatial pyramid pooling, and Adam optimizer.

Experiment 3: comparison between the proposed method with the current advanced methods. To further verify the effectiveness of the proposed approach, a comparative experiment was carried out with the other state-of-the-arts, namely AlexNet, ResNet-50, and MobileNet-V2. Based on the transfer learning (Kaya et al., 2019; Yin et al., 2020; Zhuang et al., 2021), we have considered AlexNet, ResNet-50, and MobileNet-V2 for the comparative analysis. These models were trained and injected with the weights pre-trained in the training set. In particular, in the existing network structures of Alexnet, Resnet-50 and MobileNet-V2, the original parameter weights of the models were retained and applied to the identification of pepper diseases. We have unified the dataset 224×224 to match the input dimensions of the corresponding network. To guarantee a fair compassion, the parameter settings of all the networks were kept the same. The performance indicators like average testing accuracy, average testing time, and memory requirements were used to measure the performance of the models.

Experiment 4: the recognition effect of the proposed model in the actual scene. We collected different pepper leaf diseases as the test set for the model test. The images of six different leaf diseases are 171, 170, 171, 171, 165, and 170, respectively. The 1018 pepper leaf disease images in the test set were tested by GoogLeNet-EL, AlexNet, ResNet-50, and MobileNet-V2, respectively.

### 4.4 Results analysis and discussion

**Figure 10:**
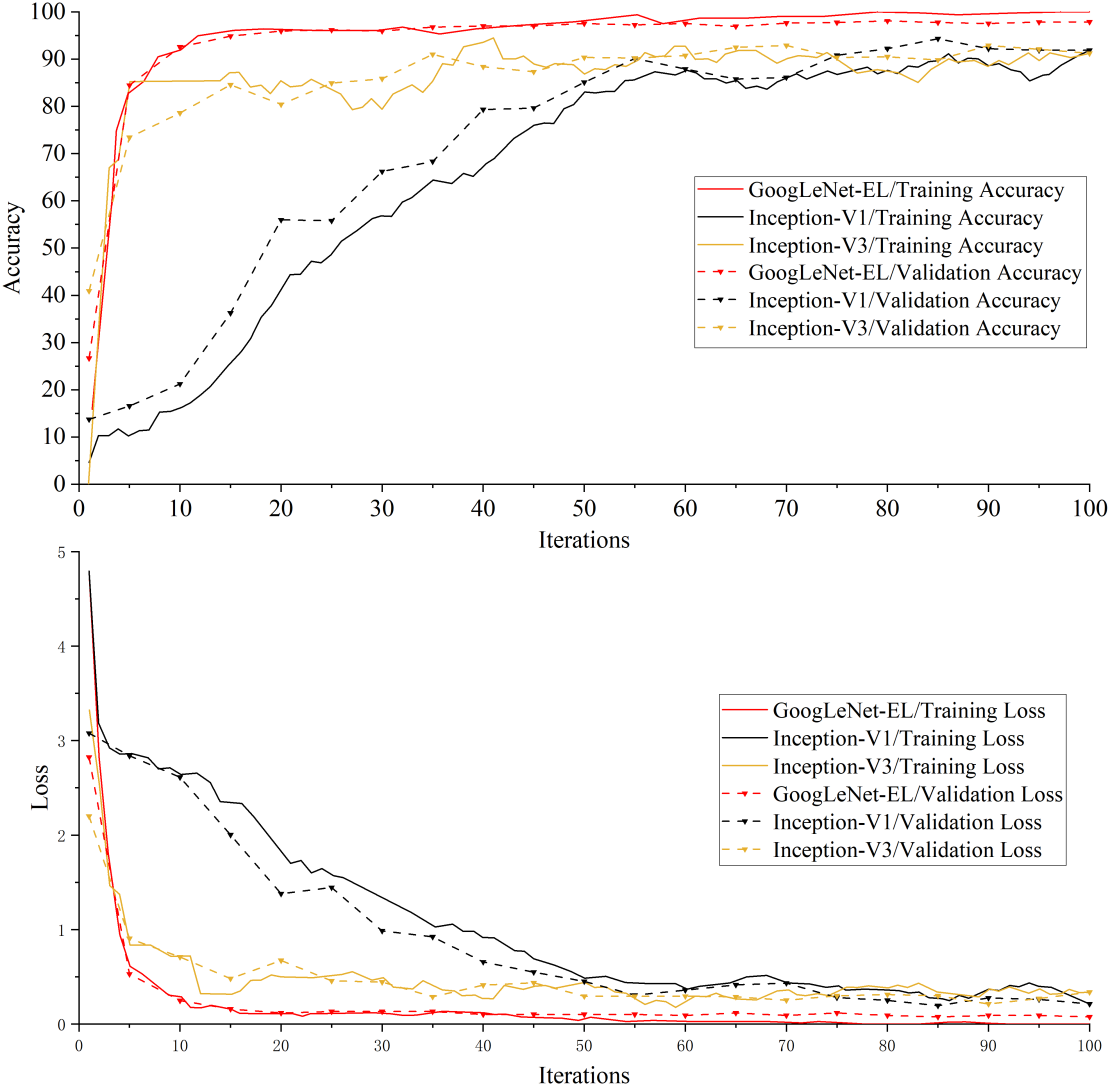
A comparison graph of the training results of the GoogLeNet-EL, Inception-V1, and Inception-V3.

Experiment 1: **Table 4** shows the experimental results of the performance indicators on the training set and validation set for the necessity test, including the accuracy, loss, training time and memory requirements. **Figure 10** is a comparison graph of the training results of GoogLeNet-EL, Inception-V1, and Inception-V3.

**Table 4:** Experimental results of different models test.

Test ID	CNN Model	Accuracy (%)	Loss (%)	Memory requirements (MB)	Training time (s)
1	GoogLeNet model (Inception-V1)	91.87	0.21	21.6	1571
2	GoogLeNet model (Inception-V3)	91.23	0.34	77.4	1646
3	Improved model (GoogLeNet-EL)	97.87	0.07	10.3	644

As shown in **Table 4** and **Figure 10**, properly simplifying the original GoogLeNet architecture can reduce the memory requirements of model training. For instance, **Table 4** shows that the memory requirement of GoogLeNet-EL is only 10.3MB, which has obvious advantages over Inception-V1 and V2 in the experiment. Due to model simplification, the calculation complexity of GoogLeNet-EL is reduced. The training time is only 644 seconds, which is significantly shorter than Inception-V1 and V2. As shown in **Figure 10**, in terms of the convergence of the accuracy curve and loss curve, within 100 iterations, the accuracy curve of GoogLeNet-EL does not fluctuate as much as that of Inception V1 and V3. Moreover, the loss curve of GoogLeNet-EL is closer to 0.

From the ablation experiment, we can observe that our improved Inception module has performed better than Inception V1 and Inception V3 regarding model size and recognition accuracy in Experiment 1. On one hand, on the basis of Inception V1, the idea of factorization of Inception V3 has been introduced to the proposed Inception module, the computational complexity has been reduced; on the other hand, the lightweight advantage of depth-wise separable convolution has been applied to the model, which can reduce the model size and improve the calculation speed without affecting the efficiency of feature extraction.

**Figure 11:**
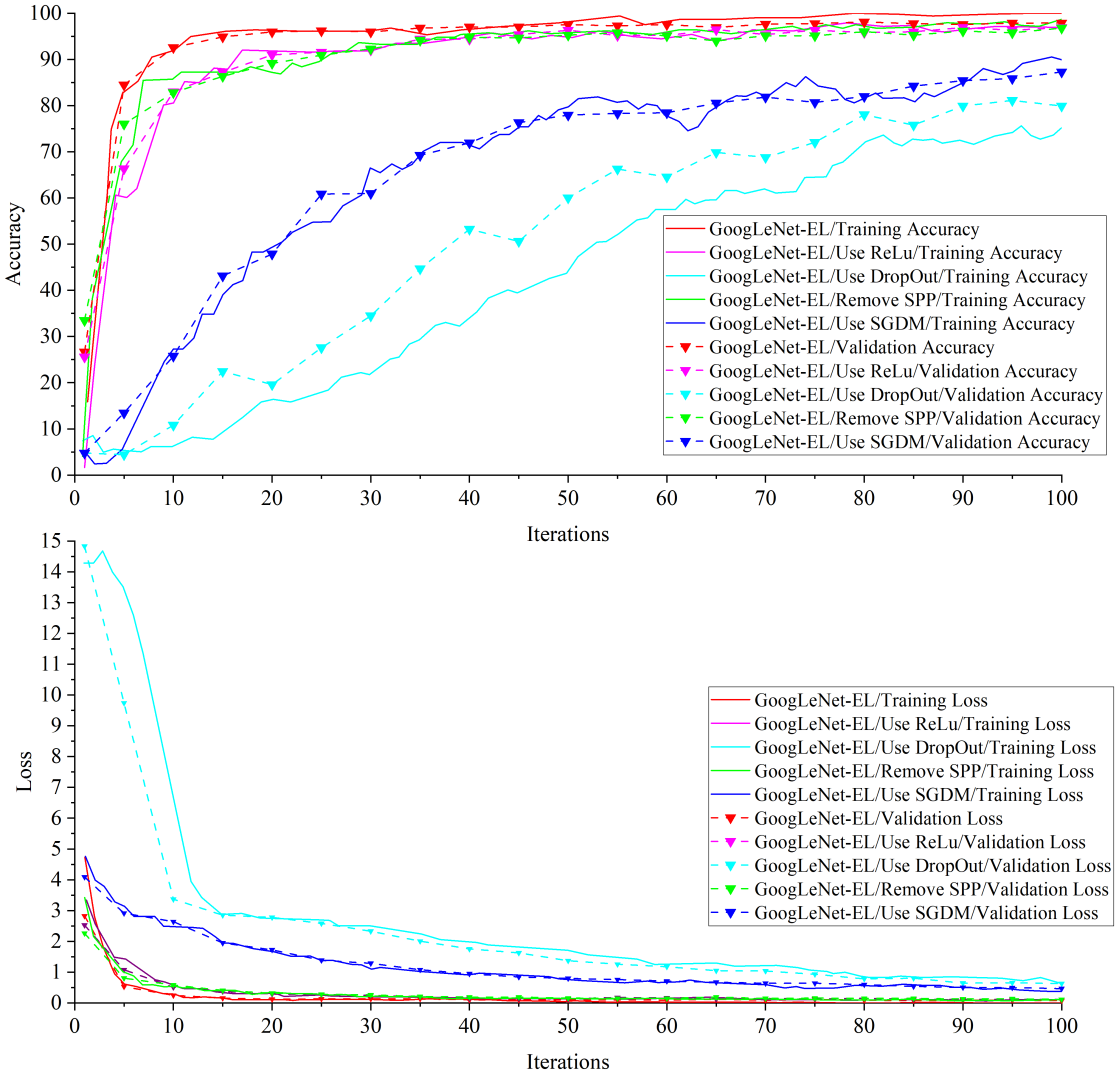
A comparison graph of the necessity test of GoogLeNet-EL related improvement aspects.

Experiment 2: **Table 5** shows the ablation experimental results of different components optimizing network structure of the proposed model. **Figure 11** is a comparison graph of the necessity test curve of the GoogLeNet-EL related improvement aspects. From the ablation experiments on four components of the model structure, we can observe the following results.

a) In the ablation experiment of ReLu vs LeakyRelu, we have investigated the impact of the activation function on model training in the GoogLeNet-EL by using ReLu and LeakyRelu, respectively. Using LeakyRelu as the activation function in terms of image convergence, **Figure 11** shows that the training accuracy curve of GoogLeNet-EL converges around 10 iterations and stabilizes at around 95%. In comparison, the training accuracy curve of ReLu as the activation function slowly converges around 15 iterations and stabilizes at around 90%. According to **Table 5**, through using LeakyRelu instead of ReLu, the accuracy and memory requirement of the model are improved by 1.01% and 0.3MB respectively. This indicates that it is effective to employ LeakyReLu as the activation function in the proposed model.
b) In the ablation experiment of DropOut, we have investigated the impact of DropOut on our proposed model. The DropOut value is set to 0.4. After using DropOut in the batch normalization layer of GoogLeNet-EL, it is seen that the accuracy curve fluctuates greatly, and there is a convergence trend within 100 iterations in **Figure 11**. Moreover, as shown in **Table 5**, the accuracy of the network training is significantly decreased, and the training time is also increased. After removing DropOut in the batch normalization layer of GoogLeNet-EL, we can observe that the accuracy and memory requirement of the model are improved by 17.96% and 0.2MB respectively. It indicates that the component DropOut could be removed.
c) In the ablation experiment of SPP, we have investigated the impact of SPP on our proposed model. As shown in **Figure 11**, after removing SPP, the training accuracy of 96.78% is lower than that of GoogLeNet-EL. The reason is that the loss of features of the target image is caused by the partially stretched image. It is proved that the introduction of SPP can bring a certain improvement to the progress of network training. In addition, SPP re-aggregates features of different dimensions into a uniform size, increasing training speed to a certain extent. It can be seen from the comparison of training time in **Table 5**.
d) In the ablation experiment of SGDM vs Adam, we have investigated the impact of SGDM and Adam on our proposed model, respectively. On the one hand, we selected SGDM as the optimizer in the GoogLeNet-EL. It can be seen from **Table 5** that the final loss value of SGDM after 100 iterations is 0.47, which is still at a relatively high level and is greatly influenced by the learning rate and other parameters. The training curve is shown in **Figure 11**. The accuracy curve is stable at 50 iterations by using SGDM. As the increase of iteration times, there is still an upward trend, but this upward trend is accompanied by an increase in training time and a great deal of fine-tuning work. On the other hand, we selected Adam as the optimizer in the GoogLeNet-EL. It can be seen that the Adam algorithm could speed up the model training and improve the convergence of model training. Therefore, it is suitable to choose Adam as the optimizer for the recognition of crop leaf diseases in our proposed model.

**Table 5:** Experimental results of different components on model performance.

Test ID	Different components description		Accuracy (%)	Loss (%)	Memory requirement (MB)	Training time (s)
1	Activation function	Use ReLu	96.86	0.11	10.6	1077
		Use LeakyReLu	97.87	0.07	10.3	644
2	Batch normalization	Use DropOut	79.91	0.63	10.5	1018
		Remove DropOut	97.87	0.07	10.3	644
3	Pooling layer	Remove SPP	96.78	0.12	10.6	915
		Use SPP	97.87	0.07	10.3	644
4	Optimizer	Use SGDM	87.28	0.47	10.6	725
		Use Adam	97.87	0.07	10.3	644

In the ablation experiment, the GoogLeNet-EL structure, which is mainly composed of LeakyReLu function, batch normalization, spatial pyramid pooling, and Adam optimizer, has performed better to recognize pepper leaf disease. It can be explained from the following perspectives: (1) In terms of activation function, compared to ReLu, the convergence rate of LeakyRelu as an activation function is faster, and the accuracy, memory requirements and training time are improved, which proves that the use of LeakyReLu as an activation function is necessary.(2) In terms of BN layer, due to the random loss of some neurons by using DropOut, the training time is greatly improved compared with InceptionV1 and V3 and the model convergence speed is accelerated, unfortunately, the training accuracy is greatly decreased. Hence, DropOut was removed in our model.(3) As for SPP, after the removal of SPP from the improved model, due to the stretching and scaling of some images, the features of the target insect pest image were lost, and the training accuracy is 96.78%, which is lower than the proposed model. However, after the adoption of SPP, there is no need to carry out size unification operation on the original image, saving some unnecessary image preprocessing time. Thus, it can improve the accuracy of the proposed model. (4) As for optimizer, Adam optimizer has obvious advantages in terms of training accuracy and loss, and the convergence effect is obviously better than that of SGDM optimizer. Thus, Adam was used instead of SGDM in our model.

**Figure 12:**
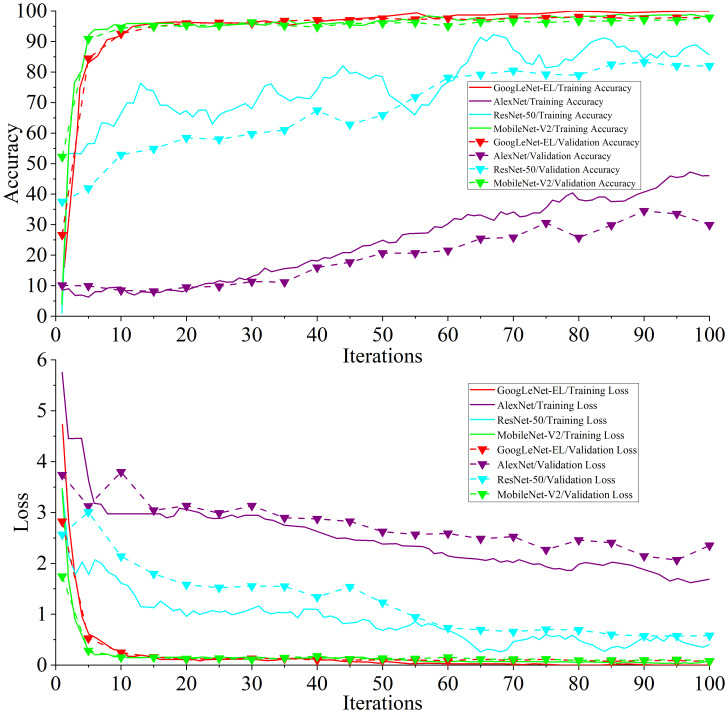
Comparison of test results with other models.

Experiment 3: **Figure 12** shows the training accuracy and loss curve compared with other models. **Table 6** shows the detailed data of the experimental result.

**Table 6:** Comparison of experimental data results with other models.

Models/methods	Accuracy(%)	Loss (%)	Memory requirement (MB)	Average testing time (ms)
AlexNet	29.92	2.35	61	50.20
ResNet-50	81.99	0.58	25.6	33.20
MobileNet-V2	96.68	0.08	8.23	25.37
Proposed method	97.87	0.07	10.3	19.33

It can be seen from **Figure 12** that the accuracy and loss curve of GoogLeNet-EL have significantly better convergence after 100 iterations, compared with AlexNet and ResNet-50. We can also observe that the validation accuracy of MobileNet-V2 and GoogLeNet-EL is the same. According to **Table 6**, we can observe that the proposed method has reached the accuracy of 97.87% and the memory requirement of 10.3MB. It is much better than what AlexNet, ResNet-50, and MobileNet-V2 could reach. In particular, the accuracy of GoogLeNet-EL is superior to that of MobileNet-V2. The memory requirement of GoogLeNet-EL is slightly inferior to the memory requirements of MobileNet-V2, but there is no significant difference between them. To summarize, it can be seen that the GoogLeNet-EL model has shown a competitive performance and obtained a superior result relative to the other state-of-the-arts. It indicates that the GoogLeNet-EL model has shown an excellent capability to recognize pepper leaf disease.

**Figure 13:**
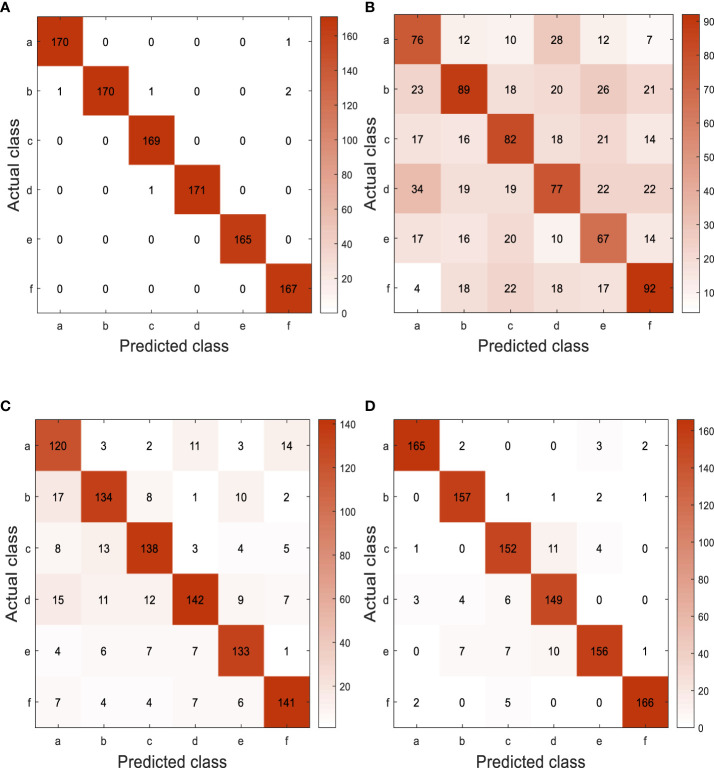
Confusion matrix for different models on test set. (a) Pepper scab, (b) Pepper powdery mildew, (c) Pepper anthracnose, (d) Pepper white spot disease, (e) Pepper blight, (f) Pepper botrytis cinerea. **(A)** GoogLeNet-EL, **(B)** AlexNet, **(C)** ResNet-50, **(D)** MobileNet-V2.

Experiment 4: **Figure 13** shows the confusion matrix of different models on the test set obtained from the final test. There are great similarities among six different types of pepper leaf diseases, so there is a phenomenon of misjudgment. According to the performance evaluation results of different models shown in **Table 7**, the accuracy, recall, and *F*1 value of GoogLeNet-EL reach about 99%, which has obvious advantages over other networks. The average testing time is 19.33ms, which is 6.04ms faster than the lightweight network MobileNet-v2; it is conducive to the further deployment of the model.

**Table 7:** Test evaluation performance of different models on the test set.

CNN model	Disease class	*Precision* (%)	*Recall* (%)	*F*1 score
GoogLeNet-EL	Pepper scab	99.4	99.4	99.4
	Pepper powdery mildew	100	97.7	98.8
	Pepper anthracnose	98.8	100	99.4
	Pepper white spot disease	100	99.4	99.7
	Pepper blight	100	100	100
	Pepper botrytis cinerea	98.2	100	99.1
AlexNet	Pepper scab	44.4	52.4	48.1
	Pepper powdery mildew	52.3	45.2	48.6
	Pepper anthracnose	48.0	48.8	48.4
	Pepper white spot disease	45.0	39.9	42.3
	Pepper blight	40.6	46.5	43.4
	Pepper botrytis cinerea	54.1	53.8	53.9
ResNet-50	Pepper scab	70.2	78.4	74.1
	Pepper powdery mildew	78.8	77.9	78.3
	Pepper anthracnose	80.7	80.7	80.7
	Pepper white spot disease	83.0	72.4	77.3
	Pepper blight	80.6	84.2	82.4
	Pepper botrytis cinerea	82.9	83.4	83.1
MobileNet-V2	Pepper scab	96.5	95.9	96.2
	Pepper powdery mildew	92.4	96.9	94.6
	Pepper anthracnose	88.9	90.4	89.6
	Pepper white spot disease	87.1	92.0	84.8
	Pepper blight	94.5	86.1	90.1
	Pepper botrytis cinerea	97.6	96.0	96.8

In addition, due to the limited data set, a cross-validation was adopted to verify the accuracy of pepper disease recognition model. In the cross-validation experiment, the pepper disease data set was randomly divided into five equal portions, where any four equal parts were used for the training set and the remaining one equal portion was used for test set. Also, the stable performance of the model was investigated under 100 iterations, and the training and testing results of the model were recorded. Under five experimental tests, the training accuracy of the model is 97.36%±1.93% and the testing accuracy of the model is 97.18%±1.93%. It demonstrates that the model has perfect accuracy and can avoid the overfitting problem.

Despite the outstanding performance demonstrated in the task of pepper leaf disease detection, there are certain limitations for further research and improvement. It was noticed that the proposed model primarily focuses on the disease areas in pepper leaf images, given its primary task of disease detection. Practical applications of the model are crucial for farmers. To make the model practically applicable for farmers, it becomes crucial to ensure its compatibility with diverse hardware platforms and software environments. Future work will explore adapting the model for use on a mobile APP. Additionally, based on the detection results, farmers expressed a desire to receive decision-making strategies to guide pesticide spraying. Hence, after identifying pepper leaf diseases, there is a plan to conduct a quantitative analysis of severity to establish a pesticide spraying model.

Moreover, the current model is mainly used for pepper disease diagnosis. We will include more diseases and crops in our future works

## 5 Conclusion

This paper has designed a convolution neural network model (GoogLeNet-EL) with high accuracy and easy transplantation for pepper leaf disease identification. Through compressing the network depth and width of the Inception module, the memory requirement of the proposed model is greatly reduced by 52.31% and 86.69% by comparing with the GoogLeNet based on Inception-V1 and Inception-V3, respectively. Experiments show that the selected LeakyReLu activation function, batch normalization algorithm, and SGDM optimizer have the best effect on the GoogLeNet-EL model and the best model fitting effect. To improve the accuracy of the model, the introduction of spatial pyramid pooling can effectively enhance the feature learning ability of the model so that the recognition accuracy of the model is 97.87%, which is more than 6% higher than that of GoogLeNet. In the real scene, we compared the proposed model with the existing mainstream models such as AlexNet, ResNet-50, and MobileNet-V2. The results show that the average testing time of the proposed model decreases by 61.49%, 41.78%, and 23.81%, respectively. In addition, the accuracy, recall, and *F*1 value of the model are about 99%, significantly higher than those network models. It demonstrates that the enhanced lightweight model has significant advantages in recognition accuracy and computing performance of pepper leaf diseases on a limited computing platform, which is beneficial to the further deployment in pepper plant in large fields.

## Data availability statement

The raw data supporting the conclusions of this article will be made available by the authors, without undue reservation.

## Author contributions

MD: Conceptualization. WS, LW, and MD: software, validation, and original draft preparation. LH, HM, SZ, XZ, and MW: review and editing, supervision. All authors improved the manuscript by responding to the review comments. All authors contributed to the article and approved the submitted version.
